# Recent advances in nanoparticles as antibacterial agent

**DOI:** 10.5599/admet.1172

**Published:** 2022-02-02

**Authors:** Murat Ozdal, Sumeyra Gurkok

**Affiliations:** Department of Biology, Science Faculty, Ataturk University, 25240 Erzurum, Turkey

**Keywords:** Nanoparticles, antimicrobials, biofilm, nanomedicine, antibiotic resistance

## Abstract

Recently, the rapid increase in antibiotic-resistant pathogens has caused serious health problems. Researchers are searching for alternative antimicrobial substances to control or prevent infections caused by pathogens. Different strategies are used to develop effective antibacterial agents, and in this respect, nanoparticles are undoubtedly promising materials. Nanoparticles act by bypassing drug resistance mechanisms in bacteria and inhibiting biofilm formation or other important processes related to their virulence potential. Nanoparticles can penetrate the cell wall and membrane of bacteria and act by disrupting important molecular mechanisms. In combination with appropriate antibiotics, NPs may show synergy and help prevent the developing global bacterial resistance crisis. Furthermore, due to characteristics such as enhanced biocompatibility and biodegradability, polymer-based nanoparticles enable the development of a wide range of medical products. Antibacterial applications of nanoparticles range from antimicrobial synthetic textiles to biomedical and surgical devices when nanoparticles are embedded/loaded/coated into different materials. In this review, the antibacterial mechanisms of nanoparticles and their potential for use in the medical field are discussed.

## Introduction

Nanoparticles (NPs) are particles in the 1-100 nm scale range. NPs have certain physical and chemical properties that differ significantly from bulk materials. Although NPs can be synthesized by different physical and chemical methods, biological synthesis is an environmentally friendly green chemistry approach that is non-toxic, biocompatible and inexpensive [1,2].

Antibiotics are very common and effective weapons in fighting infectious diseases. However, unconscious, excessive and inappropriate use of antibiotics has led to antimicrobial resistance, which is one of the serious threats affecting public health [3]. It is estimated that antimicrobial resistance may be responsible for approximately 10 million deaths per year by 2050 and will outnumber cancer deaths [4]. This situation has forced the search for new alternatives against bacterial infections. Due to high cost and various difficulties related to exploration and design, an insufficient number of novel antibiotics have been discovered in the last quarter-century [5]. With the help of nanotechnological developments, NPs that can be designed with desired properties have started to appear as promising tools. In particular, metal NPs have attracted great interest as antimicrobial agents with their unique properties. Various metal NPs including silver (Ag), copper (Cu), selenium (Se), nickel (Ni), gold (Au), zinc oxide (ZnO), titanium dioxide (TiO_2_), and iron oxide (Fe_3_O_4_), have been extensively studied for their antimicrobial effects [6].

Nanotechnology provides a new possibility for biological applications by changing the physico-chemical properties of substances. In this regard, numerous NPs have been discovered in recent years to be effective against various pathogens, including antibiotic-resistant microorganisms. The size, surface area, morphology, net charge and physicochemical properties of NPs are among the important parameters that change their antimicrobial properties through multiple mechanisms [7]. As the size of the NPs decreases, the surface-to-volume ratio increases significantly. Large surface areas of NPs provide better interaction with microorganisms and greatly influence their antimicrobial effects. Among metal NPs, positively charged ones have been reported to bind more tightly to negatively charged bacterial surfaces and show higher antimicrobial effects. The spherical NPs have been shown to have a better antimicrobial effect as they allow more ions to be released due to their larger surface area [8]. Slomberg *et al.* [9] argued that rod-like particles are more effective than spherical particles in delivering NO and inducing greater antibacterial effects across the biofilm [9]. In another study, compared with spherical or rod-shaped AgNPs, truncated triangular-shaped AgNPs with the same surface areas have been reported to have higher antibacterial activity against *Escherichia coli* [10]. Furthermore, the antibacterial action of NPs is affected by the type of capping agent used, as well as the pH and ionic strength of the medium. Capping agents adsorbed on the surface of NPs are extremely critical as stabilizers that prevent aggregation and overgrowth of NPs. These agents affect the biological activities as well as certain structural and physico-chemical properties of NPs. For example, AgNPs capped with *Solanum trilobatum* extract, a stabilizing and reducing agent, have been reported to have enhanced antimicrobial properties on Gram-negative and Gram-positive bacterial species and fungal species [11].

Bacteria use many different mechanisms to develop resistance to natural and synthetic antibiotics, rendering them ineffective and thus necessitating the development of new alternatives. However, due to the cost and complexity of developing a new antibiotic, very few new antibiotics have been commercialized in recent years. For this purpose, more attention should be paid to the development of new antimicrobial agents, which are difficult for bacteria to develop resistance against.

The aim of this review is to explain the antimicrobial mechanisms of NPs and the latest developments in antimicrobial applications.

## Mechanisms of antibacterial properties of NPs

**Figure 1.**
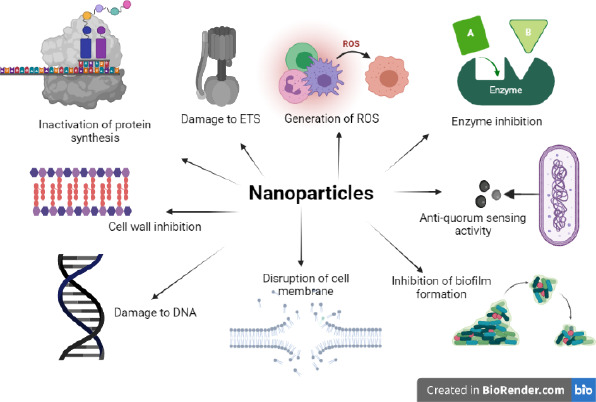
Antimicrobial mechanisms of NPs

The mechanism of the antimicrobial properties of NPs is still not fully elucidated. Some of the prominent mechanisms that cause cell death are that the particles (i) bind to the bacterial cell, impair cell membrane permeability and respiration, (ii) cause toxicity by the release of free metal ions from the surface of NPs, or (iii) cause oxidative stress by generating reactive oxygen species (ROS). Figure 1 summarizes the most proposed mechanisms. The mechanisms vary according to the NP type. Metal oxide NPs including ZnO and TiO_2_ are thought to exert bactericidal effects mostly by the generation of ROS [12], whereas Ag and Au are thought to exert bactericidal effects predominantly by releasing metal ions [13].

The lethal impact of AgNPs has been linked to the direct interaction of NPs with the bacterial cell wall, followed by penetration into the cytoplasm. NPs accumulate on the bacterial cell wall and membrane, causing morphological changes such as shrinkage of the cytoplasm, detachment of membrane, formation of multiple electron-dense pits, and eventually membrane disruption [14]. NP deposition in the cell wall of *E. coli* has been shown to create pits, which cause the release of lipopolysaccharide molecules and membrane proteins and thus the loss of outer membrane integrity, resulting in eventual cell death [14].

Metals can be chemically oxidized in aqueous solutions to give metallic ions. Due to their higher surface-to-volume ratio, NPs are able to release more ions under aerobic conditions than bulk materials. The released ions from the surface of NPs play an important role in antibacterial activity. NPs first anchor to the bacterial cell wall and, due to their nanoscale size, easily penetrate and pass through the cell wall and interact with the cell membrane. They cause structural changes in the cell membrane with the ions they release, thereby disrupting its integrity and increasing its permeability. As a result, they cause leakage of cell contents and eventually cell death. NPs targeting the cell membrane also impair membrane potential and proton motive force, block oxidative phosphorylation, and decrease intracellular ATP levels. Such an antibacterial effect of spherical AgNPs against *E. coli* was revealed by proteomic studies, which showed that exposure to AgNPs has led to the build-up of envelope protein precursors implying disruption of proton motive force [15].

Once the free metal ions reach the inside of the cell, they also interact with carbonyl, amino, phosphate and sulfhydryl (thiol) groups of the cellular biomolecules, such as DNA, proteins, and lipids [16]. It was shown that free Ag ions interact with bacterial nucleic acids preferentially via nucleosides rather than phosphate groups and degrade chromosomal DNA or interrupt the DNA replication [17]. Metal ions, such as Ag^+^, also interact with thiol groups of enzymes and proteins, altering their three-dimensional structure and blocking the active binding sites for their substrates [16]. Interference with the proteins on the cell wall and membrane disrupts the bacterial cell wall, impairing the electron transport chain inhibiting the respiratory process, and growth of the cells [18]. They interact with cytoplasmic proteins required for ATP production, inactivating them and impairing cellular functions. Ions also prevent protein synthesis by denaturing ribosomal components and interfering with the binding of ribosome subunit to tRNA [19]. Cui *et al.* [13] has shown by transcriptomic and proteomic approaches that AuNPs show antibacterial properties against *E. coli* by collapsing membrane potential and reducing ATP levels via inhibiting ATPase function and inhibiting the binding of ribosome subunit to tRNA.

Signal transduction in bacteria is another target of the antibacterial activity of NPs. Bacterial communities in a wide variety of environments are sensitive and respond to a variety of external stimuli in order to survive through their signal transduction mechanism. This mechanism, which is known to be necessary for the viability and growth of bacteria as well as the expression of virulence factors, is widely used as a target in antimicrobial drug design [20]. In this cell signaling system, a physical or chemical signal is transmitted through a cell as a cascade of molecular pathways, frequently protein phosphorylation mediated by protein kinases, which finally results in a cellular response. AgNPs 10–15 nm in size were suggested to interrupt the signal transduction by altering the phosphorylation pattern of tyrosine residues of the key proteins, which leads to apoptosis or growth inhibition in bacteria [21].

Another pivotal mechanism of the antibacterial effect of the NPs is the burst of ROS in the bacterial cell. ROS are generated during the regular oxygen metabolism and are crucial for different cellular signaling pathways. Oxygen acts as the final acceptor of electrons transported by ETS during oxidative phosphorylation and is reduced to the water molecule. Some of these electrons are taken by molecular oxygen, resulting in the formation of O^2-^, that can then be transformed into H_2_O_2_ and •OH. However, when bacterial cells are exposed to NPs, metal ions released from the surface of NPs induce ROS bursts by disrupting respiratory systems and can considerably increase intracellular ROS production. Released metal ions contribute to further increase in intracellular ROS accumulation by causing disruption of membrane integrity, inactivation of cellular enzymes, disruption of the electron transport system, and decreased membrane potentials. Bacteria have natural antioxidant defense systems to deal with oxidative stress. They have natural antioxidants like carotenes and ascorbic acid, which prevent lipid peroxidation or other ROS-related stresses. In addition, they have enzymes such as catalase, peroxidase, and superoxide dismutase that convert toxic reactive oxygen forms into non-toxic or less toxic forms. However, upon exposure to NPs, the accumulation of ROS exceeds a certain level and bacteria cannot cope with detrimental changes in vital cellular structures such as cell wall, cell membrane, DNA, and protein [7]. As a result, chemically highly reactive ROS accumulation and ROS-induced oxidative stress in the bacterial cell cause induced pore formation and lipid peroxidation in the cell membrane, damage to chromosomal DNA and proteins, and ultimately cell death. TiO_2_ and ZnO NPs show their antibacterial activities through oxidative stress. They kill microorganisms with their potent oxidizing capacity through the formation of free radicals. They are able to induce oxidative stress and DNA damage causing reduced viability of *E. coli* [12,22].

Due to the differences in bacterial cell wall structures, the effects of NPs on Gram-positive and Gram-negative bacterial species also change. In many studies, it has been reported that the antimicrobial effects of NPs on Gram-negative bacteria strains are stronger than Gram-positive bacteria. Gram-positive bacteria have a thick peptidoglycan layer consisting of many more layers, while Gram-negative bacteria have a thin peptidoglycan layer and additional lipopolysaccharides (LPS). Manzoor *et al.* [23] found that the effect of NPs was significantly more pronounced on Gram-negative strains than on Gram-positive organisms. There are also studies that suggest the opposite. Premanathan *et al.* [24] investigated the antibacterial activity of ZnO-NPs against Gram-negative *E. coli* and *Pseudomonas aeruginosa* and Gram-positive *Staphylococcus aureus* and reported that the antimicrobial effect was stronger in Gram-positive bacterium.

## Synergistic effects of NPs with antibiotics

NPs can be combined with antimicrobial agents to overcome antibiotic resistance and increase their effectiveness. In addition, they can reduce the dose and toxicity of antibiotics to be taken [5]. Since NPs act on bacteria through multiple targets and/or mechanisms, it is very difficult for the microorganism to acquire resistance. In other words, the probability of simultaneous mutations necessary for resistance formation is very low. Moreover, this is even more unlikely when NPs are combined with antimicrobials [25]. As a result, the use of NPs with antibiotics is considered as a method that can be used to prevent bacterial resistance development [26].

Antibiotics combined with NPs are more effective against both Gram-positive and Gram-negative bacteria and even drug-resistant bacteria. Aabed and Mohammed [27] showed synergistic effects of AgNPs combined with bacitracin, ciprofloxacin, tetracycline, and cefixime against *P. aeruginosa*, *E. coli*, *S. aureus*, and *Candida albicans*. Abo-Shama *et al.* [28] also demonstrated the synergistic effect of antibiotics (azithromycin, cefotaxime, cefuroxime, fosfomycin, and chloramphenicol) against *E. coli* was notably increased in the presence of AgNPs compared to the antibiotic used alone. In another study, a synergistic effect was observed when CuO NPs combined with cephalexin against *E. coli* [29].

## Anti-quorum sensing and anti-biofilm activity of NPs

Obstruction of quorum sensing (QS) is an influential alternative strategy to struggle with microbial infections. QS is a cell density-dependent regulatory process that regulates the expression of virulence factors in many pathogenic bacteria and relies on the release of extracellular small signal molecules known as autoinducers [30,31]. The QS system created by bacteria is known to be responsible for cell-to-cell communication and the formation of various virulence factors and biofilm formation [32].

Several quorum quenching (QQ) strategies have been suggested to disrupt communication between cells by degradation of a signaling molecule, inhibition of signaling molecule-receptor complex formation, inhibition of signaling molecule synthesis, inhibition of expression of QS regulated genes [33]. NPs interfere with virulence factors (pigments, enzymes, exopolysaccharides, and toxins) in bacteria with their QQ properties. García-Lara *et al.* [34] showed the inhibitory effects of ZnO NPs on QS by inhibiting the pyocyanin pigment, elastase and biofilm formation in *P. aeruginosa*. AgNPs have also been shown to inhibit the synthesis of *P. aeruginosa* virulence factors such as proteases, elastases, and pyocyanin [35,36]. In another study, Qais *et al.* [31] reported that AgNPs successfully inhibited the QS regulated virulence agents of multiple bacterial pathogens, including *P. aeruginosa*, *S. marcescens*, and *C. violaceum*.

It has also been shown that NPs effectively prevent the formation of biofilms and destroy existing ones. Biofilms, defined as well-organized multicellular microbial communities, are one of the survival strategies of microorganisms in nature. Free-floating microorganisms anchor to a surface, begin to grow, and colonize the surface. They are organized in dense clusters in the extracellular polymeric substances (EPS) they have formed [37]. This heterogeneous architecture is one of the main mechanisms that confer resistance to microorganisms against antimicrobial compounds. When bacteria form a biofilm, they become up to 1000 times more resistant to antibiotics than planktonic ones [38]. Biofilm causes undesirable conditions such as treatment failure, the persistence of infection and the development of antibiotic resistance in the clinic [39]. At least 65 % of infectious diseases (such as otitis media, lung infections, mastitis, chronic wounds, urinary tract infections, rhinosinusitis, and gingivitis) are caused by biofilm [40].

Among the antibiofilm strategies (enzymes, NPs, phages, QS inhibitors, surfactants, phyto-compounds, and antimicrobial photodynamic therapy), nanostructured materials have gained importance in recent years [41]. Ag^+^ is known to have potent antibiofilm activity and is used to prevent biofilm development on medical surfaces, including heart valves, central venous catheters, orthopedic and dental implants [35,42]. However, it is easy to sequester them with chloride and phosphate or other cellular components. NPs, on the other hand, are less exposed to sequestration due to their nano size and show a more permanent and effective antibiofilm effect. The high surface area to volume ratio, inert structure, customizable physical features such as size and shape, biocompatibility, bacteriostatic or bactericidal capabilities at extremely low concentrations are all advantages of NPs for antibiofilm applications [43]. Another advantage is that NPs are much smaller than 350 nm and can pass through biofilms. The interactions of NPs with bacteria and biofilms depend on their surface charge. Generally, positively charged NPs better penetrate biofilms [44]. This can be explained by the higher interaction of positively charged NPs with negatively charged biofilm structures (polysaccharide skeleton, proteins and DNA) and bacterial cell wall. Also, pointed and sharp triangular NPs have more vertices and ends than spherical and rod-shaped NPs, and therefore cause more damage to bacterial cells [45].

Different metallic and non-metallic NPs have been widely studied for their antibiofilm properties. The multifunctional properties of NPs make it an attractive way to enhance the effect of antimicrobial agents to control infections. Many types of NPs such as NiO [32], Ag [46], Au [47], Se, Te [48], Si [49] and AgCl-TiO_2_ [50] block the mechanism of QS and thus hinder the formation of biofilm. ZnO and TiO_2_ NPs inhibit methicillin-resistant *S. aureus* biofilm formation [51]. AgNPs synthesized by *Solibacillus isronensis* was shown to exert antibiofilm activity against *E. coli* and *P. aeruginosa* [52]. TiO_2_ nanocomposites are frequently used for surface, dental and orthopedic implant coatings [53]. The combination of AgNPs and curcumin was also found to be effective in inhibiting biofilm formation and destroying established mature biofilm [54]. Araujo *et al.* (2020) [55] studied the antimicrobial and antibiofilm effects of colloidal nanocarrier FeO NPs coated with chitosan containing chlorhexidine on *C. glabrata* and *Enterococcus faecalis* associated with oral diseases. It was reported that AuNPs coated with the antimicrobial peptide indolicidin inhibited biofilm formation [56]. Chitosan-coated FeO NPs were reported to inhibit biofilm formation in *S. aureus* [57]. When a cationic antimicrobial peptide polymyxin B was conjugated on the AgNPs surface, polymyxin B capped AgNPs showed a 3-fold higher biofilm reduction than pure AgNPs [58]. Slomberg *et al.* [9] investigated the effectiveness of nitric oxide (NO) releasing silica NPs against Gram-negative *P. aeruginosa* and Gram-positive *S. aureus* biofilms, taking into account particle size and shape. They showed that particles with reduced size and increased aspect ratio released better NO and were more effective against *P. aeruginosa* and *S. aureus* biofilms, and Gram-negative strains were more sensitive to NO. They also showed that rod-like particles were more effective than spherical particles in delivering NO and inducing greater antibacterial action across the biofilm.

## Biomedical applications of NPs

Contributing to many areas of our lives, nanotechnology has also proven to have an important potential in the field of biomedicine, and as summarized in Table 1, it has recently been used more widely. In order to prevent bacterial adhesion and biofilm formation, nano-engineering materials are developed by making surface modifications on implants and medical devices. Medical implants and devices as next-generation nanomaterials are non-expensive and biocompatible.

Antibacterial film-based composite materials enable different uses, such as implant or catheter coatings and wound dressings. When designing composite films, natural polymers such as cellulose and collagen are more preferred due to their biocompatibility. On the other hand, synthetic polymers including polyethylene, polycaprolactone, and polyurethane are used in the production of antibacterial composite films due to their durability and ease of processing [59].

There are many studies on NP-containing materials developed with antibacterial properties. The association of AgNPs with polyethylene typically reduces the wear of the polymer surface and increases the antibacterial properties of the polymer [60]. Nanocomposites consisting of a mixture of AgNPs and polyethylene can be used as agents with antimicrobial and antibiofilm inhibitory potential in the food and health fields [61]. Kim *et al.* [62] produced injectable AgNP/methylcellulose nanocomposite hydrogel for topical antimicrobial applications that can be used on burn wounds. It has been reported that a variety of antibacterial nanocomposites such as β-chitin/ZnO NPs [63], poly(vinyl alcohol)/ZnO [64], and collagen-dextran-ZnO-NPs [65] can be used to heal infected wounds. Kharaghani *et al.* [66] developed antibacterial contact lenses containing polyvinyl alcohol (PVA) AgNPs and CuNPs.

## Nanoparticles as antimicrobial agent in NP-drug conjugate system

NPs can be engineered and combined with other antimicrobial agents and they gain greater functionality in combating resistant microorganisms. Due to the chemical properties of nanoparticles, they allow long-term binding to the target site of antibiotics and protection from enzymes. Therefore, higher antibiotic requirements are avoided. It is important to create antibiotic nanoparticle conjugates to prevent multidrug-resistant pathogenic microbial infections.

The preparation of conjugated NPs is based on physical (hydrophobic, host-guest, and electrostatic interactions) and chemical interactions (with amine, trans-cyclooctene, hydrazide, isothiocyanate, sulfhydryl, azide groups of drug) [79-81]. Related possible pathways of some antibacterial nanoparticle-drug conjugate formations are presented below.

- The nanoparticle antibiotic combination provides great benefits in the solubility of poorly soluble drugs, drug half-life, systemic circulation, and drug release.
- The negative charge of peptidoglycan layer, lipopolysaccharides, and teichoic acid promotes adhesion of NPs and makes bacteria more sensitive to antimicrobial therapy.
- Hydrogenation of NPs increases the stability of NPs and impairs their function by binding to the negatively charged surface of the bacterial cell.
- By binding to the proteins in the bacterial cell membrane, the NPs increase the permeability and more antibiotics are passed into the bacterial cell. The active surface of NPs causes membrane damage, disrupts protein–protein interactions and metabolic disorders in cells.
- NPs interact with sulfhydryl (-SH) groups in the cell wall to form R-S-S-R bonds and inhibit respiration resulting in cell death. When NPs enter bacteria, they can affect cell membrane functions (permeability, respiration).

Since multiple simultaneous mutations are required in the same microorganism, antimicrobial resistance is unlikely to develop if antibiotics are combined with NPs. There are still many unexplored conjugates. Therefore, new antimicrobials are waiting to be discovered.

## Pharmacology and Toxicity of Nanoparticles

The pharmacokinetics of NPs depends on various factors such as physicochemical properties (morphology, composition, size, charge, etc.), route of exposure (topical, intramuscular, intradermal, parenteral and subcutaneous), dosage, and animal species. Absorption of AuNP, AgNP or TiO_2_ NPs is usually low by the oral, dermal or pulmonary route. Inhalational absorption of AuNPs ranges from 0.06% to 5.5% and oral absorption is about 0.01–5% for AuNPs, 1–4.2% for AgNPs, and 0.01–0.05% for TiO_2_ NPs depending on the size [82-83]. Excessive accumulation of NPs in the target tissue is generally desired for therapeutic effects, and conversely, high levels of distribution or accumulation to non-target tissues can result in undesirable toxicity. Further research is needed to understand better the pathway and dose differences in the pharmacokinetics of NPs.

Contrary to the positive aspects of nanoparticles, they can cause adverse effects on living things. Nanoparticles have found use in many industrial areas such as food, cosmetics, medicine, textiles, and automotive with their extraordinary physicochemical properties. In parallel with their increasing use in daily life, humans are constantly exposed to nanoparticles by inhalation, oral, dermal contact, and intravenous injection routes and NPs can pose a potential threat in long-term exposures.

The interaction of NPs with body tissues and thus their toxic effect has not yet been fully elucidated. It is predicted that NPs may have varying effects according to their type and physicochemical properties, exposure route, dose and time, and type of the cell line [84]. The toxicity of Ag NPs has been extensively studied in vitro and Ag NPs have been demonstrated to be more harmful to cell lines than other metal NPs [85]. It has been observed that as the size of the NP increase, the adverse health effects also increase [86]. NPs injected intravenously can deposit in the colon, liver, spleen, and lymphatic system [87]. Inhalation of NPs might cause cytotoxicity in the lung [88].

Once in the body, NPs can easily enter bloodstream due to their small size and be transported to other parts of the body, such as the lungs, liver, kidneys, and reproductive organs. Here, they can accumulate and interact with tissues leading cytotoxicity and dysfunction of the organs. Moreover, due to their very small size, NPs can even cross the blood-brain barrier and cause neurotoxicity [89]. Another concern of using the antimicrobial properties of NPs is the possibility that they can cause the death of beneficial human microflora.

NPs are known to be cytotoxic, carcinogenic, genotoxic, apoptosis inducer, and cell proliferation inhibitor [90]. Negative effects caused by NPs in living things usually occur by the destruction of cell membranes and organelles [91] or by binding to biomacromolecules and changing their structures and functions [92].

The toxicity of NPs has been recognized by numerous in vivo studies. Repeated 28-day oral exposure of albino Wistar rats to magnesium oxide (MgO) NPs caused damage to DNA, chromosomes, proteins and enzymes, redox balance and increased hepatic enzyme concentration in blood [93]. It has been reported in a study with rats that oxidative stress caused by CuO NP interacts with cell components and induces hepatotoxicity and nephrotoxicity [94]. Ag-NPs and TiO_2_-NPs affect the central nervous system and cause neuroinflammation by inducing glial cell activation to release proinflammatory cytokines and produce ROS and nitric oxide [89]. Cytotoxic effects of AgNPs on osteoclasts and osteoblasts, the cardiovascular and respiratory systems, DNA, and embryo development abnormalities, have been studied. NPs can also cause hemolysis and disrupt the blood coagulating mechanisms [68].

Since the side effects of NPs are not fully understood, their use in clinical applications is limited and more studies are needed to benefit from NPs more effectively. Clarifying the toxicity of NPs with detailed in vivo and clinical studies will pave the way for the routine use of NPs in combating infections caused by multi-drug resistant bacteria. To reduce the harmful effects of NPs on organisms, there are different approaches, such as the use of antioxidants. The beneficial effects of using antioxidant substances have been reported, especially in toxicities associated with NP-induced oxidative stress formation [95]. In addition, proper adjustment of the threshold dose and exposure time that inhibit cell viability is vital for the safe delivery of NPs.

## Conclusions

Nanotechnology offers alternative sources to antibiotics. In order to struggle the infections by bacteria, nanoparticles exhibit multiple features such as inhibition of biofilms and/or increased intracellular accumulation of NPs. The nanotechnological intervention provides new possibilities for the development of new therapeutic drug candidates to control the QS-regulated virulence profile, biofilm formation, and drug resistance profile. When nanoparticles are used together with appropriate antibiotics against pathogens, they reduce the amount of antibiotics to be applied, minimizing both the possibility of resistance development and toxicity. This synergistic effect of nanoparticles with antibiotics can be used against pathogenic bacteria in the near future. Recent advances in nanotechnology have enabled the synthesis of new nanomaterials with multifunctional antimicrobial properties. When nanoparticles are embedded/loaded/coated into/on different materials, they can be used in a variety of applications, from antimicrobial synthetic textiles to biomedical and surgical equipment. Biopolymer-based nanomaterials allow the development of many medicinal products due to their advantages, such as increased biocompatibility and biodegradability. In the light of increasing research in the field of nanomedicine, new uses of antibacterial nanoparticles will be revealed.

## Figures and Tables

**Table 1.** Different nanomaterials used in biomedical fields with antibacterial properties

Biomaterials	Potential applications	Bacteria	Reference
Cotton/silk fabrics containing reduced graphene oxide (RGO) and Ag/Cu NPs	Antimicrobial protective medical textiles	*P. aeruginosa* *E. coli* *S. aureus*	[67]
Polyvinyl alcohol containing Ag/Cu NPs	Antibacterial contact lenses	*S. aureus* *P. aeruginosa*	[66]
Lysozyme-coated AuNPs in combination with the β-lactam	Diabetic wound healing	*S. aureus* *Acinetobacter calcoaceticus* *P. aeruginosa* *E. coli* *Klebsiella pneumoniae* *Bacillus subtilis*, B. cereus	[68]
Keratin containing AgNPs	Skin wound healing and tissue recovery	*E. coli* *S. aureus*	[69]
AgNPs-loaded bacterial cellulose hydrogels	Moist wound-healing hydrogels	*S. aureus* *P. aeruginosa*	[70]
Colloidal NPs (ZnO, CuO, TiO_2_ Ag)	Antiseptic mouthwashes	*S. mutans* *Streptococcus sangius*	[71]
Dextran/CeO_2_ NPs	Against implant infections	*P. aeruginosa* *S. epidermidis*	[72]
Collagen conjugated with AgNP	Repairing of infected bone	Vancomycin-resistant *S. aureus*	[73]
AgNPs with alginate-nano hydroxyapatite	Potential candidate for bone tissue repair and regeneration	*S. aureus*	[74]
Ag–ZnO@ carboxymethyl cellulose/K-carrageenan/graphene oxide/konjac glucomannan hydrogel	Nursing care for diabetic foot ulcers	*S. aureus* *E. coli*	[75]
Collagen - chondroitin sulfate - fibronectin - AgNPs	Oral cavity lesions repair	*Fusobacterium nucleatum* *Porphyromonas gingivalis*	[76]
Pyrolytic carbon coated with AgNP	Antibacterial artificial heart valve	*MRSA* *S. pyogenes* *E. coli* *K. pneumoniae* *P. aeruginosa, Proteus vulgaris*	[77]
Face mask coated with colloidal AgNPs	Antimicrobial face masks	*S. aureus* *E. coli*	[78]
